# The *sinR* Ortholog PGN_0088 Encodes a Transcriptional Regulator That Inhibits Polysaccharide Synthesis in *Porphyromonas gingivalis* ATCC 33277 Biofilms

**DOI:** 10.1371/journal.pone.0056017

**Published:** 2013-02-06

**Authors:** Reiko Yamamoto, Yuichiro Noiri, Mikiyo Yamaguchi, Yoko Asahi, Hazuki Maezono, Masae Kuboniwa, Mikako Hayashi, Shigeyuki Ebisu

**Affiliations:** 1 Department of Restorative Dentistry and Endodontology, Osaka University Graduate School of Dentistry, Suita, Osaka, Japan; 2 Department of Preventive Dentistry, Osaka University Graduate School of Dentistry, Suita, Osaka, Japan; Charité-University Medicine Berlin, Germany

## Abstract

Biofilm-forming cells are distinct from well characterized planktonic cells and aggregate in the extracellular matrix, the so-called extracellular polymeric substances (EPS). The *sinR* gene of *Bacillus subtilis* encodes a transcriptional regulator that is known to be involved in the biosynthesis of EPS in biofilms. *Porphyromonas gingivalis* inhabits the subgingival and extraradicular biofilm of humans and is one of the primary pathogens that cause progressive marginal and refractory apical periodontitis. Furthermore, *P. gingivalis* possesses PGN_0088, which encodes a putative ortholog of *B. subtilis sinR*. Here, we investigated the role of PGN_0088 (*sinR*) on biofilm formation. *P. gingivalis* strains formed biofilms on saliva-coated glass surfaces in phosphate buffered saline. Quantitative analysis indicated that the biofilm of the *sinR* null mutant consisted of dense exopolysaccharide. Microscopic observations showed that the increased levels of exopolysaccharide produced by the *sinR* mutant changed the morphology of the EPS to a mesh-liked structure. Furthermore, physical analyses suggested that the enrichment of exopolysaccharide in the EPS enhanced the resistance of the biofilm to hydrodynamic shear force. The results presented here demonstrate *sinR* plays important roles in the ability of *P. gingivalis* strain ATCC 33277 to act as a negative mediator of exopolysaccharide accumulation and is indirectly associated with the structure of the EPS and the force of its adhesion to surfaces.

## Introduction

Bacteria adhere widely to surfaces of diverse composition in the environment. These biofilms cause problems in a number of activities, such as agriculture, industry, and healthcare [1]. In the dental field, oral biofilms are defined to consist of multiple bacterial species and to cause opportunistic infection, resulting in dental caries and periodontal disease [2], [3]. *Porphyromonas gingivalis*, a Gram-negative oral anaerobe, is distributed throughout subgingival and extraradicular biofilms and is one of the major pathogens in severe forms of marginal periodontitis and refractory periapical periodontitis [4], [5]. Subgingival biofilms localize to the gingival sulcus, pathologically called the periodontal pocket, with thicknesses that range from tens to hundreds of microns [6]. An extraradicular area is located outside the apex field of a root canal over the apical foramen, and biofilms of 30 to 40 µm thickness are known to occupy the extraradicular area of patients with refractory periapical periodontitis [7].

Generally, the properties of bacteria in biofilms are markedly different from those in their planktonic state [8]. These changes occur in response to a variety of environmental signals and are reflected in the new phenotypic characteristics of biofilm-forming cells [9]. In the biofilm, cells aggregate in the EPS that they generate [10]. In most microorganisms, the EPS occupies more than 90% of the dry mass, forms the scaffold for the 3-dimensional (3D) architecture of the biofilm, and is responsible for adhesion to surfaces and for cohesion in the biofilm [11]. The EPS protects organisms against desiccation, oxidizing or charged biocides, some antibiotics and metallic cations, ultraviolet radiation, many protozoan grazers, and host immune defenses [11]. Therefore, deciphering the mechanism of matrix production might be lead to the development of a novel method for controlling the formation of biofilms.

In biofilms harboring the spore-forming bacterium *Bacillus subtilis*, the EPS consists of an exopolysaccharide, which is specified by the *epsA–O* operon and a secreted protein TasA, which is encoded by the *yqxM-sipW-tasA* operon [12]. The *epsA–O* and *yqxM-sipW-tasA* operons are controlled by the repressor protein SinR [13]–[15]. In contrast, using microarray analysis, we revealed that the number of genes differentially regulated by more than 1.5-fold was highest at the later stage of biofilm formation by *P. gingivalis* strain ATCC 33277 (312/2,090 genes) [16]. Among them, PGN_0088 (one of the orthologs of *sinR*) was the most highly down-regulated (3.59-fold) gene. PGN_0088 and it's ortholog of *B. subtilis*, *sinR*, are 41.4% identical (http://www.kegg.jp/ssdb-bin/ssdb_best?org_gene=pgn:PGN_0088).

PGN_0088 (*sinR*) is listed in GenBank as a putative transcriptional regulator (http://www.ncbi.nlm.nih.gov/gene/6330436) and its actual biological function remains to be defined. Here, we demonstrated that the *sinR* ortholog PGN_0088 inhibits polysaccharide synthesis and infects the structure of EPS in *P. gingivalis* ATCC 33277 biofilms. We further showed that the mutation of *sinR* induced resistance to physical disruption, owing to the high exopolysaccharide per cell ratio.

## Results

### Quantitative analysis of protein and carbohydrates in biofilm of the *sinR* mutant

**Figure 1:**
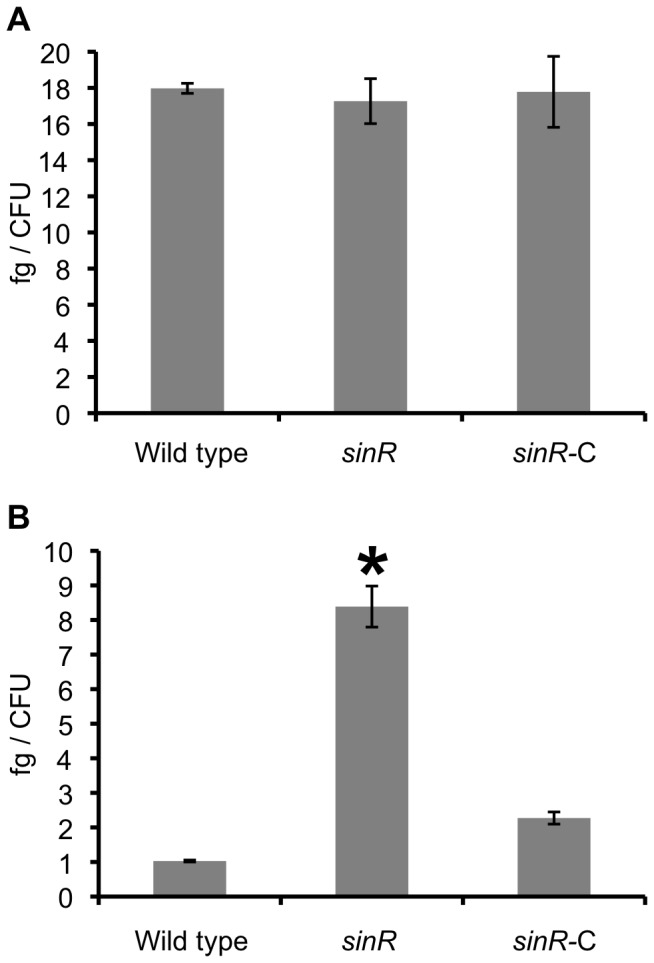
Quantification of components of biofilms formed by *P. gingivalis* strains. *P. gingivalis* strains were incubated in PBS for 24 h. After washing, the amounts of protein (A) and carbohydrate (B) per CFU were determined using the colorimetric methods described in the Methods section. Statistical analysis was performed using a Welch's t test. *P<0.001 in comparison with the wild type strain.

We measured the amounts of protein and carbohydrate in biofilms on saliva-coated coverglasses using BCA protein assay kit and the phenol-sulfuric acid method. There was no significant difference in the amounts of protein per colony formation unit (CFU) between wild type, *sinR* mutant strain (*sinR*), and *sinR* ^+^-complemented strain (*sinR*-C) (Figure 1A). In contrast, the biofilm formed by *sinR* contained significantly larger amounts of carbohydrate per CFU than biofilms formed by the wild type and *sinR* ^+^-C (Figure 1B).

### Confocal laser scanning microscope (CLSM) observation of biofilms formed by the *sinR* mutant

**Figure 2:**
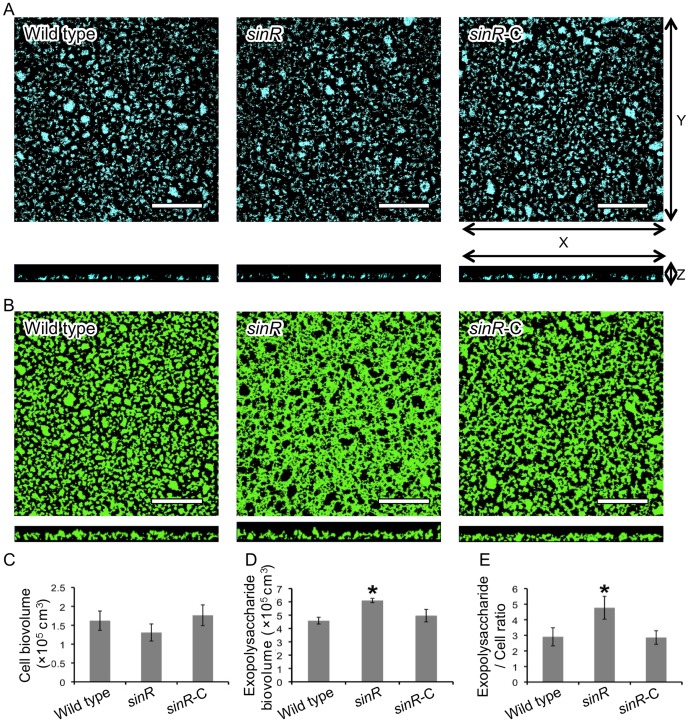
CLSM observation of biofilms formed by *P. gingivalis* strains. *P. gingivalis* strains were stained with DAPI (blue) and incubated in PBS for 24 h. After washing, exopolysaccharide was stained with FITC-labeled concanavalin A and wheat germ agglutinin (green). *P. gingivalis* cells (A) and exopolysaccharides (B) of biofilms that developed on the coverglasses were observed with a CLSM equipped with a 40× objective. Scale bars represent 50 µm. Optical sections were obtained along the z-axis at 0.7-µm intervals, and images of the x-y and x-z planes were reconstructed with imaging software as described by Kuboniwa *et al*. [19]. Fluorescent images were quantified using Imaris software and the average of total cell biovolume per field (C) and that of total exopolysaccharide biovolume per field (D) were calculated. Furthermore, exopolysaccharide levels are expressed as the ratio of exopolysaccharide/cells (FITC/DAPI) fluorescence (E). The experiment was repeated independently three times. Data are presented as average of 8 fields per sample along with the standard errors of the mean. Statistical analysis was performed using a Welch's t test. *P<0.001 in comparison with the wild type strain.

We evaluated the structure of the *sinR* mutant biofilm on the saliva-coated coverglasses using CLSM. The image of 4′,6-diamino-2-phenylindole (DAPI)-labeled cells of *sinR* could not be distinguished from wild type or *sinR*-C (Figure 2A). In contrast, only the image of fluorescein isothiocyanate (FITC)-labeled exopolysaccharide of *sinR* showed a mesh-like structure (Figure 2B). Quantitation of the images did not detect a significant difference in the cell biovolumes among the examined three strains (Figure 2C), whereas the biovolume and average substratum coverage of the exopolysaccharide of *sinR* was significantly larger than those of the other two strains (Figures 2D and S1D). Furthermore, exopolysaccharide production was normalized to the levels of cells in the biofilms and expressed as the exopolysaccharide per cell ratio. The ratio of the exopolysaccharide per cell of *sinR* was significantly higher than those of the other strains (Figure 2E).

### Scanning electron microscopy (SEM) of the biofilm produced by the *sinR* mutant

**Figure 3:**
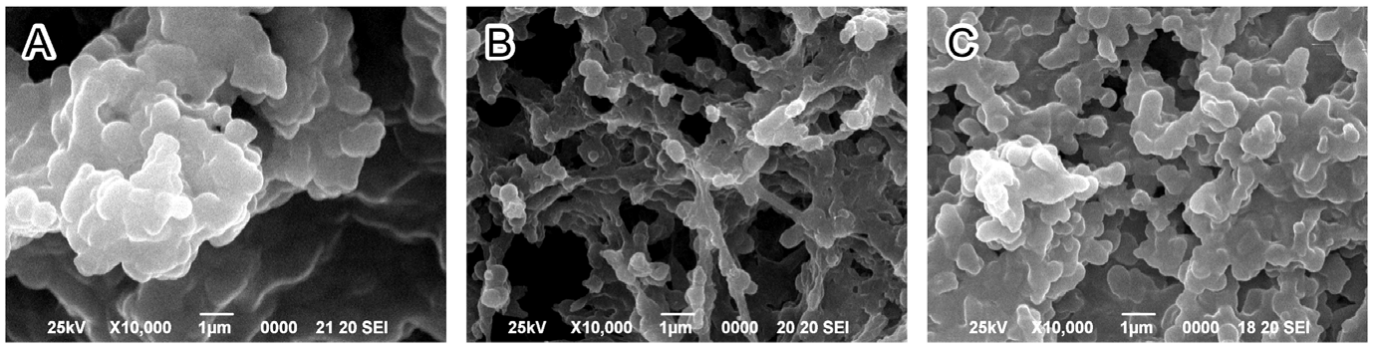
SEM observation of biofilms formed by *P. gingivalis* strains. *P. gingivalis* wild type (A), *sinR* mutant (*sinR*, B) and *sinR* ^+^-complemented (*sinR*-C, C) strains formed biofilms that developed on the coverglasses were observed with a SEM.

We examined the surface structure of the *sinR* biofilm on the saliva-coated coverglasses using SEM. The EPS-like structure of wild type and *sinR*-C strain biofilms exhibited a flattened shape (Figure 3A and 3C) in contrast to that of *sinR*, which was mesh-like (Figure 3B).

### Physical strength of biofilm of *sinR* mutant

**Figure 4:**
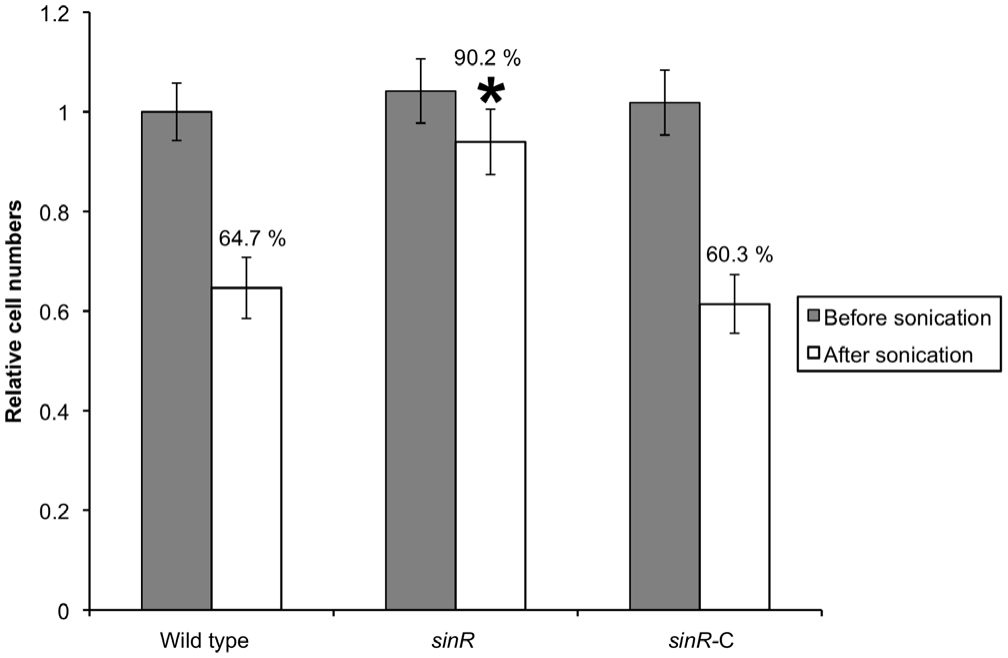
Strength of biofilms formed by *P. gingivalis* strains. Standardized cultures of *P. gingivalis* were inoculated into dGAM in saliva-coated 12-well polystyrene plate and incubated statically at 37°C for 60 h, and the resulting biofilms were sonicated for 1 s. Immediately after sonication, supernatants containing floating cells were removed by aspiration and the biofilm remnants were gently washed with PBS. *P. gingivalis* genomic DNA was isolated from the biofilms, and the numbers of *P. gingivalis* cells were determined using real-time PCR. Relative amounts of bacterial cell numbers were calculated based on the number of wild type cells without sonication and assigned a value of 1.0. The percentages shown indicate the amount of remaining biofilm after sonic disruption. Duplicate experiments were independently repeated three times with each strain. Standard error bars are shown. Statistical analysis was performed using Welch's *t* test. *P<0.001 in comparison with the wild type strain.

To analyze the influence of the mutation of the *sinR* gene on the stability of biofilms, we compared the mutant's ability to resist brief ultrasonication and found that it was significantly higher resistant to sonic disruption than the other two strains (Figure 4).

## Discussion

Microorganisms synthesize the EPS present in their biofilms [10]. The EPS that protects organisms against biocides, and host immune defenses is widely recognized as one of the main reasons that biofilms cause a number of problems, such as intractability of infection and failure of treatment [11]. In *B. subtilis*, *sinR* controls the biosynthesis of the EPS [13]. Furthermore, *P. gingivalis* possesses PGN_0088 as one of the orthologs of *sinR* of *B. subtilis*. In our present study, we muted PGN_0088 (*sinR*) and investigated the role of this gene in the formation of biofilms formed by *P. gingivalis* strain ATCC 33277.

The amount of carbohydrate in *P. gingivalis* biofilms was reduced by the expression of SinR (Figures 1 and 2). Furthermore, the mature biofilm of *sinR* mutant formed by using the flow-cell model described in our previous publication [16] contained significantly more carbohydrate than that of wild type. In *B. subtilis*, SinR acts on the *epsA–O* operon as a transcriptional regulator and depresses the biosynthesis of exopolysaccharide in biofilms [17]. *P. gingivalis* has at least three sugar macromolecules on its surface as follows: lipopolysaccharide (LPS), anionic cell surface polysaccharide (APS), and capsular polysaccharide (CPS). APS functions to anchor arginine-specific gingipain A (RgpA) on the bacterial outer membrane and is distinct from LPS and CPS [18], [19]. Acting as a transcription factor, SinR could participate in the regulation of the expression of some of these polysaccharides.

In *B. subtitlis* SinR also controls the *yqxM-sipW-tasA* operon whose products participate in the biosynthesis of a secreted protein, TasA [13]. In the present study, the SinR of *P. gingivalis* decreased overall levels of carbohydrate but not that of proteins (Figure 1). An important group of biofilm matrix-associated proteins are those that polymerize into fibers variously known as pili or fimbriae [20], [21]. *P. gingivalis* produces long (FimA) and short (Mfa) fimbriae [19]. In our previous study, expression of fimbriae-associated genes during the development of biofilms was elevated in the early stage but remained unchanged during the later stages [16]. Furthermore, expression of *sinR* was down-regulated only in the late stage of biofilm formation. In the present study our focus was on the transcriptional behavior of *sinR*, and studies on protein expression will be performed next. Moreover, in our present study, we only measured the total amount of protein. Thus, it is remain unresolved if the SinR protein influences the production of fimbriae. Further work on the influence of SinR on the expression of individual proteins containing fimbriae is necessary to define the targets of its activity.

Our present study demonstrates that SinR has an inhibitory effect on synthesis of exopolysaccharide in *P. gingivalis* biofilms. Therefore, we also determined the influence of carbohydrate levels on the morphological and physicochemical properties of biofilms formed by *P. gingivalis*. The EPS of bacterial biofilms comprises exopolysaccharides, proteins, lipids, nucleic acids, lipoteichoic acids, and lipopolysaccharides [11], [22]–[25]. The individual components of the EPS vary dynamically according to local environmental conditions [11], [25], [26]. Studies of diverse bacterial species have revealed that change in the quantity of any of these components influences the 3D-structure of EPS. For example, biofilms of a fimbriae-deficient strain (*flp*-1-disrupted mutant) of the periodontal pathogen *Aggregatibacter actinomycetemcomitans* forms microcolonies in looser formation, and fibrils of fimbriae are not observed [27]. Furthermore, its adhesion to the surface was significantly blocked by sodium metaperiodate or DNase I treatment but not by proteases. This mutant secretes carbohydrates and DNA instead of fimbriae to coalescent on a surface [27]. Friedman and Kolter screened for transposon insertion mutants of *Pseudomonas aeruginosa* PA14 that were unable to form pellicles that represent one type of biofilm formed at the air-liquid interface in static cultures [28]. They identified 7 flanking genes (*pel*) that contribute to the formation of the pellicle, and revealed that the products of these genes are involved in the construction of the EPS [28]. In *B. subtilis*, the structures of the biofilms formed by the *eps* (required for production of exopolysaccharide) mutant and *tasA* (forms amyloid fibers) mutant were flat. In contrast, the biofilms produced by the *sinR* (inhibitor of *eps* and *tasA*) mutant were extremely wrinkly [19], [21]. The CLSM (Figure 2B) and SEM (Figure 3) images acquired in the present study show that the mutation of *sinR* induces morphological changes of the EPS from a laminar to a mesh-like structure. Thus, the SinR produced by *P. gingivalis* ATCC 33277 might be indirectly involved in the 3D-conformation of the EPS in biofilms by controlling the expression of genes associated with the EPS components.

*Xylella fastidiosa*, a bacterium responsible for Pierce's disease in grapevines, possesses both type I and type IV pili at the same cell pole. De La Fuente *et al*. [29] evaluated the attachment of the bacteria to a glass substratum using a microfluidic flow chamber in conjunction with pilus-defective mutants. The adhesion force required to disperse *X. fastidiosa* mutant possessing only type I pili was significantly higher, whereas that of mutant cells possessing only type IV pili was significantly lower than those of wild type cells [29]. In contrast, Kuboniwa *et al*. [19] revealed that the exopolysaccharide per cell ratio of biofilms formed by a *fimA* mutant was significantly higher than that of wild type and that the mutant formed a tough and cohesive biofilm. Furthermore, the exopolysaccharide per cell ratio of the biofilm formed by an arginine-specific gingipain A and B (RgpA and RgpB, respectively) double-mutant was significantly smaller than that of wild type, and the biofilm of the mutant was fragile. Hence, in *P. gingivalis*, the exopolysaccharide per cell ratio might correlate with resistance to physical disruption.

We also show here that the *sinR* mutant formed carbohydrate-rich and stout biofilms (Figure 4). The exopolysaccharide of the *P. gingivalis* biofilm could contribute to the adhesion force to the surface; however, further studies are required to demonstrate that this is the case. Mounting evidence has accumulated over the past 20 years that supports a role for *P. gingivalis* in periodontal disease and infection and as a potential risk factor for several systemic diseases, including diabetes, preterm birth, heart disease, and atherosclerosis [30]–[34]. Dispersal of bacteria from the biofilm at the periodontal pocket or extraradicular area facilitates spread throughout the body via the bloodstream and initiates or detrimentally influences these systemic conditions [32]. Many factors trigger bacterial Dispersal from biofilms, including alterations in the availability of nutrients (such as carbon sources), oxygen depletion, low levels of nitric oxide, changes in temperature, and high or low levels of iron [35]. Furthermore, bacteria possess regulatory systems to drive differential gene expression in response to these changing conditions [35]. Acquisition of resistance to physical disruption by deletion of *sinR* might be associated with tolerance against the dispersal of biofilm. In the future, uncovering the relationship between *sinR* and biofilm dispersal will lead to the development of a method to block the diffusion of *P. gingivalis* from its biofilm.

## Conclusions

Our investigations here on the role of *sinR* on the production during the formation of biofilms by *P. gingivalis* strain ATCC 33277 show that the gene is a negative mediator of exopolysaccharide synthesis and influences with the 3D-structure of the EPS. Furthermore, the biofilm produced by the *sinR* mutant exhibited resistance to physical disruption, owing to the high exopolysaccharide per cell ratio. These findings suggest that research focused on defining the detailed mechanisms of biofilm formation by *P. gingivalis* will contribute to the development of more effective methods for preventing pathologies associated with bacterial biofilms.

## Materials and Methods

### Bacterial strains and culture conditions

All bacterial strains used in this study are shown in Table 1. *P. gingivalis* was grown anaerobically (10% CO_2_, 10% H_2_, and 80% N_2_) in Gifu anaerobic medium (GAM; Nissui, Tokyo, Japan) broth [17] on enriched tryptic soy agar [36], [37]. For selection and maintenance of antibiotic-resistant strains, antibiotics were added to the medium at the following concentrations (µg/ml): ampicillin, 100; erythromycin, 10; gentamycin, 200 or tetracycline, 0.7.

**Table 1:** Bacterial strains, plasmids, and primers used in this study.

Strains, Plasmids and Primers	Description	Source
***E. coli***		
S17-1	*thi*,*pro*,*hsdR*-,*hsdM*+,*recA*; integrated plasmid RP4-Tc::Mu-Kn::Tn7	[40]
***P. gingivalis***		
33277	Wild type	ATCC
ODP001	*sinR::ermF*	This study
ODP002	*sinR::ermF* pTCB-*sinR*	This study
**Plasmids**		
pBluescript® II SK(−)	Amp^r^, cloning vector	Stratagene
pGEM®-T Easy	Amp^r^, PCR TA cloning vector	Promega
pOD001	Amp^r^, contains the *sinR*-upstream and downstream region of KpnI-NotI-digested pBluescript® II SK(−)	This study
pOD002	Amp^r^, contains the *ermF* within the *Bam*HI-digested fragment of pOD001	This study
pKD355	Amp^r^ Erm^r^, contains the *ermF ermAM* DNA block of pVA2198 [44] between *Eco*RI and *Bam*HI of pUC18	[38]
pOD003	Amp^r^, contains the *sinR* region in pGEM®-T Easy	This study
pOD004	Amp^r^, *tetQ*, contains the *sinR* region within *Not*I-*Bam*HI-digested pTCB	This study
pTCB	Amp^r^, *tetQ*, contains the MCS of pBluescript® II KS within *Ava*I-*Hind*III-digested pT-COW	[39]
**Primers**		
SUF	5′-GATCCTCGTAAAAAGCACGAGTATCGTAT-3′	
SDR	5′-GGTACCTGCTACTACTACCTGCACGACATT-3′	
SDF	5′-GCGGCCGCATGCAGGAAACGAGAAGAGATT-3′	
SDR	5′-GGATCCAGCAGCCTTCTCTTGAGATGCTAT-3′	
SCF	5′-GCGGCCGCTATATCCCAATGTCATCCAACG-3′	
SCR	5′-GGATCCGGAATCGGAAATCCTTACCTTTAT-3′	
SSF	5′-CAATGTCAGCTTGACTGGTAATACT-3′	
SSR	5′-AGGAGGATTAAGGATGGAACTATTG-3′	
EMF	5′-ATGACAAAAAAGAAATTGCCCG-3′	
EMR	5′-CTACGAAGGATGAAATTT-3′	

### Construction of bacterial strains and plasmids

**Figure 5:**
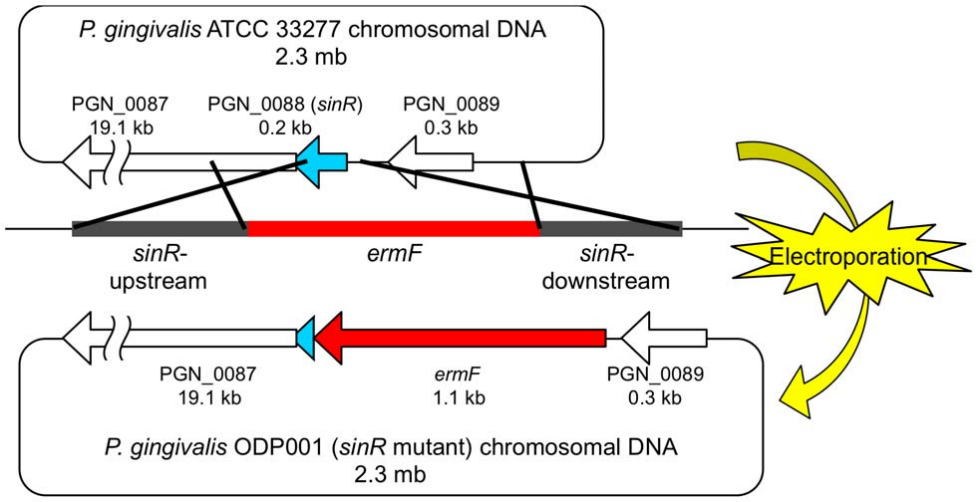
Scheme of construction of the *sinR* mutants. To yield strain ODP001 (*sinR* mutant; Δ*sinR::ermF*), the linearized DNA fragment including *ermF* (red) was introduced into chromosomal DNA of *P. gingivalis* ATCC 33277 by electroporation.

**Figure 6:**
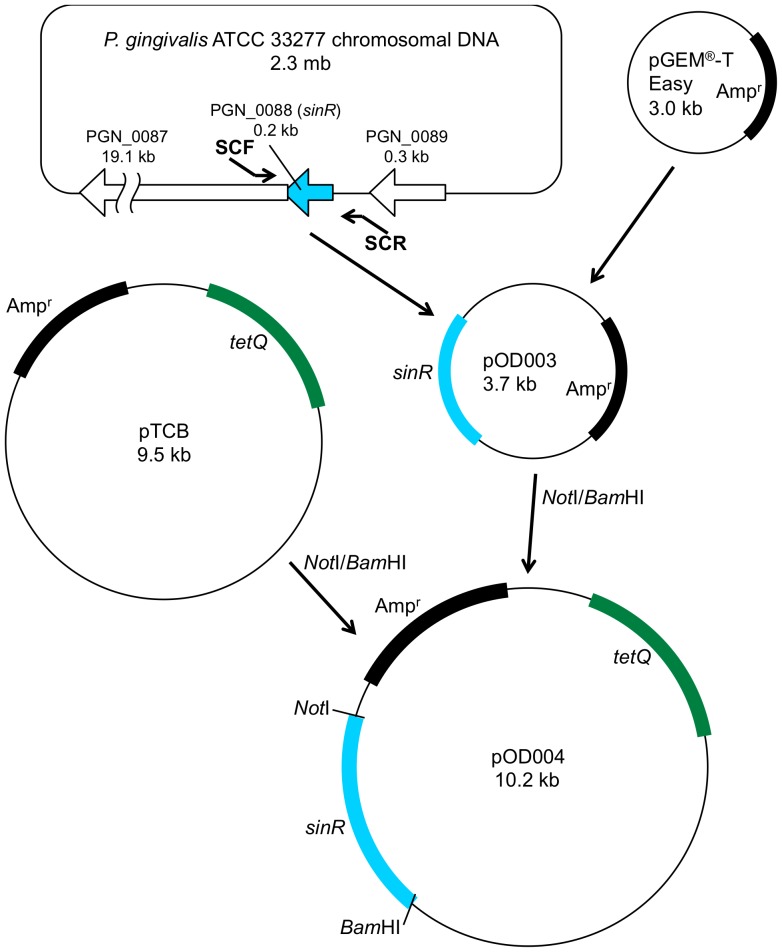
Scheme of construction of the *sinR* ^+^-complementing strain. The 0.7-kb DNA fragment (aqua) containing the *sinR* gene region was PCR-amplified from ATCC 33277 chromosomal DNA with SCF and SCR primers. The amplified DNA fragment was cloned into the pGEM®-T Easy vector, resulting in pOD003. The *sinR* region DNA obtained by *Not*I and *Bam*HI digestion was inserted into *Not*I-*Bam*HI-digested pTCB [38] to yield pOD004 (*sinR* ^+^).

A *P. gingivalis* PGN_0088 (*sinR*) deletion mutant was constructed according to the method of Yamaguchi *et al* [37] as follows: *sinR*-upstream and *sinR*-downstream DNA regions were amplified using the polymerase chain reaction (PCR) of strain ATCC 33277 chromosomal DNA with the primer pair SUF and SUR for the *sinR*-upstream region and with the primer pair SDF and SDR for the *sinR*-downstream region. The DNA primers and plasmids used in this study are listed in Table 1. The amplified DNA fragments were digested with *Kpn*I and *Bam*HI to generate the *sinR*-upstream region and with *Bam*HI and *Not*I to generate the *sinR*-downstream region, which was then inserted into *Kpn*I-*Not*I–digested pBluescript® II SK(−) (Stratagene, La Jolla, CA.) to yield pOD001. The 1.1-kb *Bam*HI-digested *ermF* DNA cartridge was acquired from pKD355 [38] using *Bam*HI-digestion and inserted into the *Bam*HI site of pOD001, resulting in pOD002 (Δ*sinR::ermF*). The *Bss*HII-linearized pOD002 DNA fragment was introduced into *P. gingivalis* ATCC 33277 by electroporation (Figure 5) using a Gene-Pulser Xcell Microbial System (Bio-Rad Laboratories, Richmond, CA.) set at 25 µF, 400 Ω, and 2.5 kV to yield strain ODP001 (*sinR* mutant; Δ*sinR::ermF*). To construct the *sinR* ^+^-complementing strain (Figure 6), the 0.7-kb DNA fragment containing the *sinR* gene region was amplified from ATCC 33277 chromosomal DNA using PCR primed by SCF and SCR. The amplified DNA fragment was cloned into the pGEM®-T Easy vector (Promega, Madison, WI.), resulting in pOD003. The *sinR* region DNA obtained by *Not*I and *Bam*HI digestion was inserted into *Not*I-*Bam*HI-digested pTCB [39] to yield pOD004 (*sinR* ^+^). The pOD004 plasmid DNA was introduced into the *sinR* mutant by conjugation with *E. coli* S17-1 [40] harboring pOD004 as a donor strain, resulting in strain ODP002 (*sinR* ^+^-complemented strain; Δ*sinR::ermF/sinR* ^+^). Ampicillin, erythromycin, gentamycin or tetracycline was used to select colonies, which harbored these antibiotic-resistant gene cassettes.

### DNA probes and Southern blot hybridization

A DNA fragment (0.8 kb) comprising the *sinR* gene was amplified using PCR from 33277 chromosomal DNA with the primer pair SSF and SSR. An *ermF* DNA fragment (0.8 kb) was from pKD355 [38] using PCR with the primer pair EMF and EMR [37]. These fragments were labeled with the AlkPhos Direct system for chemiluminescence (GE Healthcare, Amersham, UK). Southern blotting was performed using a nylon membrane and hybrids were detected using the CDP-star reagent. The results of this analysis confirmed that the *ermF* DNA cartridge was inserted into the PGN_0088 locus of ODP001.

### Analysis of biofilm formation

#### Quantitative analysis

Biofilms were formed on a chambered coverglass system (Lab-Tek™ Chambered Coverglass; Nalge Nunc International, Rochester, NY.). Human saliva, centrifuged at 2,000× *g* for 15 min and then filter-sterilized using a syringe filter with a pore size of 0.22 µm (Millex-GP Filter Unit, SLGP033RB; Millipore, Billerica, MA) [16], was poured on wells of the coverglass and incubated overnight. *P. gingivalis* cells were washed and suspended in phosphate-buffered saline (PBS) at an optical density at 550 nm (OD_550_) of 1.0, then cultured in saliva-coated wells of the coverglass for 24 h.

For quantitative analysis, biofilms were washed and resuspended in 1 mL PBS. Protein and carbohydrate concentrations of biofilm suspensions were determined using the BCA protein assay kit (Thermo Scientific, Rockford, IL.) according to the manufacturer's protocols and the phenol-sulfuric acid method as described previously [41]. Next, the numbers of colony forming units (CFUs) were calculated by determining the copy numbers of the *P. gingivalis* ATCC 33277 16S rRNA gene in biofilms essentially according to the method of Kuboniwa *et al.* [19], [42]. The amounts of protein and carbohydrate per cell were calculated by dividing their concentrations by CFU equivalents.

#### CLSM observation

Biofilms were formed on the chambered coverglass described above. Briefly, *P. gingivalis* cells were stained with DAPI (50 µg/ml; Molecular Probes, Eugene, OR.), washed, suspended in PBS at an OD_550_ of 1.0, and cultured in saliva-coated wells of the coverglass for 24 h. The resulting biofilms were washed and the exopolysaccharide was labelled with Concanavalin-A-FITC conjugate and Wheat germ agglutinin-FITC as described previously [19]. After washing, images were obtained using CLSM (LSM-510; Carl Zeiss, München-Hallbergmoos, Germany) with reflected laser light at 405 and 488 nm and then analyzed as described above. Eight images per fields per a sample were acquired. The experiment was independently repeated three times.

#### SEM observation and quantitative analysis

Biofilms were formed as described above for the quantitative analyses. The resulting biofilms were washed, treated, and observed using SEM as described by Yamaguchi *et al.* [37] and Asahi *et al*. [43].

#### Sonic disruption assay

This assay was performed essentially according to the method of Kuboniwa *et al*. [19]. Briefly, 24-well tissue culture plates (Becton Dickinson Labware, Franklin Lakes, NJ) were coated with human saliva. *P. gingivalis* cells (1.5×10^9^ CFU/well) were statically incubated in diluted GAM (dGAM; GAM/PBS ratio, 1∶4) for 60 h at 37°C, and the resulting biofilms were sonicated for 1 second at output level 1 (output power, 25 W; oscillating frequency, 28 kHz; tip diameter, 2.5 mm) with a Handy Ultrasonic Disruptor (UR-20P, Tomy Seiko, Tokyo, Japan). Genomic DNA was isolated from biofilms formed by *P. gingivalis* and the CFU value (see above) was determined using real-time PCR, as described previously [19], [42]. The data represent the mean ± standard error of the mean of three separate experiments performed in duplicate for each strain.

### Statistical analysis

The significance of intergroup differences of all data was analyzed using Welch's *t* tests. A *P* value<0.001 was considered to indicate statistical significance.

## Supporting Information

Figure S1 **Quantification of mean thickness and average substratum coverage from CLSM observation.** Fluorescent images of CLSM (Figures 2A and 2B) were quantified using Imaris software and the mean thickness of cells (A) and that of exopolysaccharide (B), and average substratum coverage of cells (C) and that of exopolysaccharide (D) per field were calculated. The experiment was repeated independently three times. Data are presented as average of 8 fields per sample along with the standard errors of the mean. Statistical analysis was performed using a Welch's t test. *P<0.001 in comparison with the wild type strain.
(TIF)

## References

[1] Costerton JW, Cheng KJ, Geesey GG, Ladd TI, Nickel JC, et al (1987) Bacterial biofilms in nature and disease. Annu Rev Microbiol 41: 435–464. 3318676 10.1146/annurev.mi.41.100187.002251

[2] Costerton JW, Stewart PS, Greenberg EP (1999) Bacterial biofilms: a common cause of persistent infections. Science 284: 1318–1322. 10334980 10.1126/science.284.5418.1318

[3] Donlan RM, Costerton JW (2002) Biofilms: survival mechanisms of clinically relevant microorganisms. Clin Microbiol Rev 15: 167–193. 11932229 10.1128/CMR.15.2.167-193.2002 PMC118068

[4] Noiri Y, Li L, Yoshimura F, Ebisu S (2004) Localization of *Porphyromonas gingivalis*-carrying Fimbriae in situ in Human Periodontal Pockets. J Dent Res 83: 941–945. 15557402 10.1177/154405910408301210

[5] Noguchi N, Noiri Y, Narimatsu M, Ebisu S (2005) Identification and localization of extraradicular biofilm-forming bacteria associated with refractory endodontic pathogens. Appl Environ Microbiol 71: 8738–8743. 16332869 10.1128/AEM.71.12.8738-8743.2005 PMC1317348

[6] Noiri Y, Ozaki K, Nakae H, Matsuo T, Ebisu S (1997) An immunohistochemical study on the localization of *Porphyromonas gingivalis*, *Campylobacter rectus* and *Actinomyces viscosus* in human periodontal pockets. J Periodontal Res 32: 598–607. 9401932 10.1111/j.1600-0765.1997.tb00937.x

[7] Noiri Y, Ehara A, Kawahara T, Takemura N, Ebisu S (2002) Participation of bacterial biofilms in refractory and chronic periapical periodontitis. J Endod 28: 679–683. 12398163 10.1097/00004770-200210000-00001

[8] Costerton JW, Geesey GG, Cheng KJ (1978) How bacteria stick. Sci Am 238 Feb: 86–95. 635520 10.1038/scientificamerican0178-86

[9] O'Toole G, Kaplan HB, Kolter R (2000) Biofilm formation as microbial development. Annu Rev Microbiol 54: 49–79. 11018124 10.1146/annurev.micro.54.1.49

[10] Costerton JW, Lewandowski Z, Caldwell DE, Korber DR, Lappin-Scott HM (1995) Microbial biofilms. Annu Rev Microbiol 49: 711–745. 8561477 10.1146/annurev.mi.49.100195.003431

[11] Flemming HC, Wingender J (2010) The biofilm matrix. Nat Rev Microbiol 8: 623–633. 20676145 10.1038/nrmicro2415

[12] Chu F, Kearns DB, McLoon A, Chai Y, Kolter R, et al (2008) A novel regulatory protein governing biofilm formation in *Bacillus subtilis* . Mol Microbiol 68: 1117–1127. 18430133 10.1111/j.1365-2958.2008.06201.x PMC2430766

[13] Chu F, Kearns DB, Branda SS, Kolter R, Losick R (2006) Targets of the master regulator of biofilm formation in *Bacillus subtilis* . Mol Microbiol 59: 1216–1228. 16430695 10.1111/j.1365-2958.2005.05019.x

[14] Winkelman JT, Blair KM, Kearns DB (2009) RemA (YlzA) and RemB (YaaB) regulate extracellular matrix operon expression and biofilm formation in *Bacillus subtilis* . J Bacteriol 191: 3981–3991. 19363116 10.1128/JB.00278-09 PMC2698397

[15] Lopez D, Vlamakis H, Kolter R (2009) Generation of multiple cell types in *Bacillus subtilis* . FEMS Microbiol Rev 33: 152–163. 19054118 10.1111/j.1574-6976.2008.00148.x

[16] Yamamoto R, Noiri Y, Yamaguchi M, Asahi Y, Maezono H, et al (2011) Time course of gene expression during *Porphyromonas gingivalis* strain ATCC 33277 biofilm formation. Appl Environ Microbiol 77: 6733–6766. 21803908 10.1128/AEM.00746-11 PMC3187161

[17] Kearns DB, Chu F, Branda SS, Kolter R, Losick R (2005) A master regulator for biofilm formation by *Bacillus subtilis* . Mol Microbiol 55: 739–749. 15661000 10.1111/j.1365-2958.2004.04440.x

[18] Paramonov N, Rangarajan M, Hashim A, Gallagher A, Aduse-Opoku J, et al (2005) Structural analysis of a novel anionic polysaccharide from *Porphyromonas gingivalis* strain W50 related to Arg-gingipain glycans. Mol Microbiol 58: 847–863. 16238632 10.1111/j.1365-2958.2005.04871.x

[19] Kuboniwa M, Amano A, Hashino E, Yamamoto Y, Inaba H, et al (2009) Distinct roles of long/short fimbriae and gingipains in homotypic biofilm development by *Porphyromonas gingivalis* . BMC Microbiol 9: 105. 19470157 10.1186/1471-2180-9-105 PMC2697998

[20] Epstein EA, Chapman MR (2008) Polymerizing the fibre between bacteria and host cells: the biogenesis of functional amyloid fibres. Cell Microbiol 10: 1413–1420. 18373633 10.1111/j.1462-5822.2008.01148.x PMC2674401

[21] Romero D, Aguilar C, Losick R, Kolter R (2010) Amyloid fibers provide structural integrity to *Bacillus subtilis* biofilms. Proc Natl Acad Sci U S A 107: 2230–2234. 20080671 10.1073/pnas.0910560107 PMC2836674

[22] Karatan E, Watnick P (2009) Signals, regulatory networks, and materials that build and break bacterial biofilms. Microbiol Mol Biol Rev 73: 310–347. 19487730 10.1128/MMBR.00041-08 PMC2698413

[23] Ma L, Conover M, Lu H, Parsek MR, Bayles K, et al (2009) Assembly and development of the *Pseudomonas aeruginosa* biofilm matrix. PLoS Pathog 5: e1000354. 19325879 10.1371/journal.ppat.1000354 PMC2654510

[24] Mann EE, Rice KC, Boles BR, Endres JL, Ranjit D, et al (2009) Modulation of eDNA release and degradation affects *Staphylococcus aureus* biofilm maturation. PLoS One 4: e5822. 19513119 10.1371/journal.pone.0005822 PMC2688759

[25] Xiao J, Klein MI, Falsetta ML, Lu B, Delahunty CM, et al (2012) The Exopolysaccharide Matrix Modulates the Interaction between 3D Architecture and Virulence of a Mixed-Species Oral Biofilm. PLoS Pathog 8: e1002623. 22496649 10.1371/journal.ppat.1002623 PMC3320608

[26] Branda SS, Vik S, Friedman L, Kolter R (2005) Biofilms: the matrix revisited. Trends Microbiol 13: 20–26. 15639628 10.1016/j.tim.2004.11.006

[27] Inoue T, Shingaki R, Sogawa N, Sogawa CA, Asaumi J, et al (2003) Biofilm formation by a fimbriae-deficient mutant of *Actinobacillus actinomycetemcomitans* . Microbiol Immunol 47: 877–881. 14638999 10.1111/j.1348-0421.2003.tb03454.x

[28] Friedman L, Kolter R (2004) Genes involved in matrix formation in *Pseudomonas aeruginosa* PA14 biofilms. Mol Microbiol 51: 675–690. 14731271 10.1046/j.1365-2958.2003.03877.x

[29] De La Fuente L, Montanes E, Meng Y, Li Y, Burr TJ, et al (2007) Assessing adhesion forces of type I and type IV pili of *Xylella fastidiosa* bacteria by use of a microfluidic flow chamber. Appl Environ Microbiol 73: 2690–2696. 17293518 10.1128/AEM.02649-06 PMC1855618

[30] Genco RJ (1996) Current view of risk factors for periodontal diseases. J Periodontol 67: 1041–1049. 10.1902/jop.1996.67.10.1041 8910821

[31] Morrison HI, Ellison LF, Taylor GW (1999) Periodontal disease and risk of fatal coronary heart and cerebrovascular diseases. J Cardiovasc Risk 6: 7–11. 10197286 10.1177/204748739900600102

[32] Li X, Kolltveit KM, Tronstad L, Olsen I (2000) Systemic diseases caused by oral infection. Clin Microbiol Rev 13: 547–558. 11023956 10.1128/cmr.13.4.547-558.2000 PMC88948

[33] Dasanayake AP, Russell S, Boyd D, Madianos PN, Forster T, et al (2003) Preterm low birth weight and periodontal disease among African Americans. Dent Clin North Am 47: 115–125. 12519009 10.1016/s0011-8532(02)00056-3

[34] Gibson FC III, Yumoto H, Takahashi Y, Chou HH, Genco CA (2006) Innate immune signaling and *Porphyromonas gingivalis*-accelerated atherosclerosis. J Dent Res 85: 106–121. 16434728 10.1177/154405910608500202

[35] McDougald D, Rice SA, Barraud N, Steinberg PD, Kjelleberg S (2012) Should we stay or should we go: mechanisms and ecological consequences for biofilm dispersal. Nat Rev Microbiol 10: 39–50. 10.1038/nrmicro2695 22120588

[36] Nakayama K, Kadowaki T, Okamoto K, Yamamoto K (1995) Construction and characterization of arginine-specific cysteine proteinase (Arg-gingipain)-deficient mutants of *Porphyromonas gingivalis*. Evidence for significant contribution of Arg-gingipain to virulence. J Biol Chem 270: 23619–23626. 7559528 10.1074/jbc.270.40.23619

[37] Yamaguchi M, Sato K, Yukitake H, Noiri Y, Ebisu S, et al (2010) A *Porphyromonas gingivalis* mutant defective in a putative glycosyltransferase exhibits defective biosynthesis of the polysaccharide portions of lipopolysaccharide, decreased gingipain activities, strong autoaggregation, and increased biofilm formation. Infect Immun 78: 3801–3812. 20624909 10.1128/IAI.00071-10 PMC2937442

[38] Ueshima J, Shoji M, Ratnayake DB, Abe K, Yoshida S, et al (2003) Purification, gene cloning, gene expression, and mutants of Dps from the obligate anaerobe *Porphyromonas gingivalis* . Infect Immun 71: 1170–1178. 12595429 10.1128/IAI.71.3.1170-1178.2003 PMC148816

[39] Nagano K, Murakami Y, Nishikawa K, Sakakibara J, Shimozato K, et al (2007) Characterization of RagA and RagB in *Porphyromonas gingivalis*: study using gene-deletion mutants. J Med Microbiol 56: 1536–1548. 17965357 10.1099/jmm.0.47289-0

[40] Simon R, Priefer U, Plihler A (1983) A broad host range mobilization system for in vivo genetic engineering: transposon mutagenesis in gram-negative bacteria. Bio/Technology 1: 784–794.

[41] Hodge JE, Hofreiter BT (1962) Determination of reducing sugars and carbohydrates. In: Whistler RL, Wolfrom ML, editors. Methods in Carbohydrate Chemistry. Volume 1: Academic Press. pp. 380–394.

[42] Kuboniwa M, Amano A, Kimura KR, Sekine S, Kato S, et al (2004) Quantitative detection of periodontal pathogens using real-time polymerase chain reaction with TaqMan probes. Oral Microbiol Immunol 19: 168–176. 15107068 10.1111/j.0902-0055.2004.00135.x

[43] Asahi Y, Noiri Y, Igarashi J, Suga H, Azakami H, et al (2012) Synergistic effects of antibiotics and an N-acyl homoserine lactone analog on *Porphyromonas gingivalis* biofilms. J Appl Microbiol 112: 404–411. 22093286 10.1111/j.1365-2672.2011.05194.x

[44] Fletcher HM, Schenkein HA, Morgan RM, Bailey KA, Berry CR, et al (1995) Virulence of a *Porphyromonas gingivalis* W83 mutant defective in the *prtH* gene. Infect Immun 63: 1521–1528. 7890419 10.1128/iai.63.4.1521-1528.1995 PMC173184

