# Supplementary figures and images for: The sinR Ortholog PGN_0088 Encodes a Transcriptional Regulator That Inhibits Polysaccharide Synthesis in Porphyromonas gingivalis ATCC 33277 Biofilms
Source: PLoS One. 2013 Feb 6;8(2):e56017. doi: 10.1371/journal.pone.0056017 (PMC3566044; doi:10.1371/journal.pone.0056017)

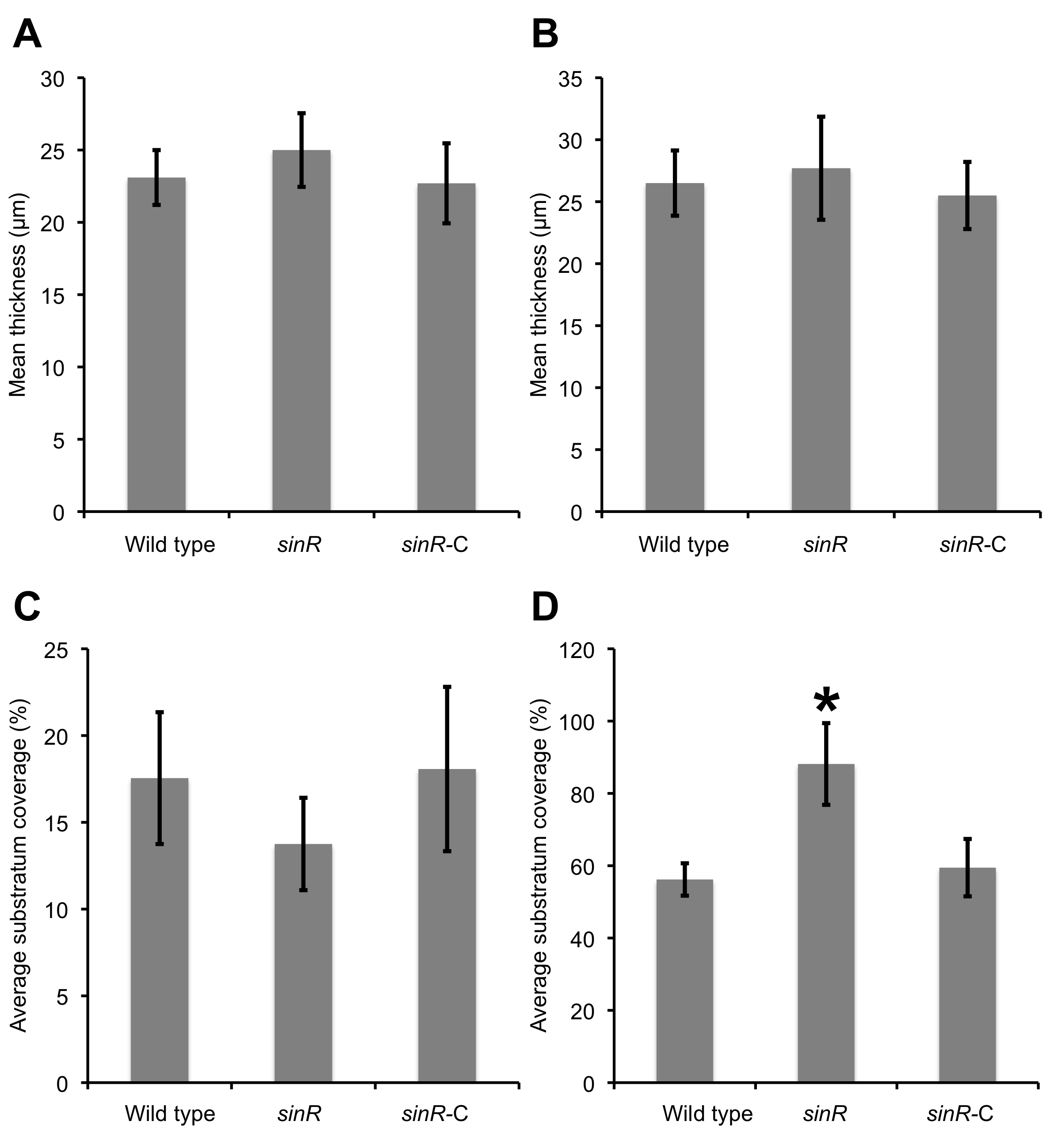

Supplement: Figure S1 — Quantification of mean thickness and average substratum coverage from CLSM observation. Fluorescent images of CLSM (Figures 2A and 2B) were quantified using Imaris software and the mean thickness of cells (A) and that of exopolysaccharide (B), and average substratum coverage of cells (C) and that of exopolysaccharide (D) per field were calculated. The experiment was repeated independently three times. Data are presented as average of 8 fields per sample along with the standard errors of the mean. Statistical analysis was performed using a Welch's t test. *P<0.001 in comparison with the wild type strain. (TIF) [file pone.0056017.s001.tif]
